# Effect of substrate stiffness on early human embryonic stem cell differentiation

**DOI:** 10.1186/1754-1611-7-7

**Published:** 2013-03-21

**Authors:** Nikolai Eroshenko, Rukmani Ramachandran, Vamsi K Yadavalli, Raj R Rao

**Affiliations:** 1 Department of Chemical and Life Science Engineering, Virginia Commonwealth University, Richmond, VA, USA; 2 Current Address: School of Engineering and Applied Sciences, Harvard University, Boston, MA, USA

**Keywords:** Stem cells, Biomaterials, Extracellular matrix, Differentiation, Stiffness

## Abstract

**Background:**

The pluripotency and self renewing properties of human embryonic stem cells (hESC) make them a valuable tool in the fields of developmental biology, pharmacology and regenerative medicine. Therefore, there exists immense interest in devising strategies for hESC propagation and differentiation. Methods involving simulation of the native stem cell microenvironment, both chemical and physical, have received a lot of attention in recent years. Equally important is evidence that cells can also sense the mechanical properties of their microenvironment. In this study, we test the hypothesis that hESCs accept mechanical cues for differentiation from the substrate by culturing them on flexible polydimethylsiloxane (PDMS) of varying stiffness.

**Results:**

PDMS substrates were prepared using available commercial formulations and characterized for stiffness, surface properties and efficiency of cell attachment and proliferation. Across different substrate stiffness, cell numbers, cell attachment and cell surface area were found to be similar. Expression of pluripotency markers decreased with increased time in culture across all PDMS substrates of varying stiffness. Analysis of gene expression of differentiation markers indicates that the differentiation process becomes less stochastic with longer culture times.

**Conclusions:**

We evaluated the utility of PDMS substrates for stem cell propagation and substrate mediated differentiation. The stiffness affected gene expression of pluripotent and differentiation markers with results indicating that these substrate systems could potentially be used to direct hESC fate towards early mesodermal lineages. This study suggests that coupled with soluble factors, PDMS substrates could potentially be useful in generating defined populations of differentiated cells.

## Background

Human embryonic stem cells (hESCs) are characterized by their ability to self renew and to differentiate into any diploid human cell type. This property makes them a valuable tool for studying the basic biology of lineage specification, and for applications in fields such as pharmacology and tissue engineering [1]. The control of stem cell fate has chiefly been attributed to genetic specifications and cellular response to signals from the surrounding niche in the form of chemical, mechanical and matrix factors [2]. Multiple studies have shown that soluble factors such as fibroblast growth factors (FGFs), bone morphogenetic proteins (BMPs) and Wnts can regulate stem cell behaviour [3-6] and have been used as chemical cues in methodologies to generate clinically relevant cell populations. Mammalian cells also generate and are exposed to forces *in vivo* and *in vitro*; and these forces can influence stem cell fates by modulating cell shape, cytoskeletal structure and interaction with the extra cellular matrix (ECM) [7,8]. Simulating in-vivo mechanical deformations by imposing substrate strains has been shown to influence stem cell differentiation and such responses to mechanical loading depend not only on the type of stem cell but also on the state of differentiation and the type of strain applied [9-11]. Such studies indicate that even the mechanical properties of the culture system may be modified and tailored to generate a desired cell population. Despite these numerous efforts, the creation of efficient, reliable, and scalable differentiation protocols has remained largely elusive.

Recent data suggesting that the extracellular matrix influences stem cell fate has led to interest in research involving control of stem cell fate by directing ECM geometry/ topography, mechanical properties, transmission of mechanical and biophysical factors to the cell, and the control of cell geometry [12,13]. In most cases, individual stem cells do not survive in suspension and their adhesion to a matrix is therefore essential for viability. The ECM as a major niche element provides not only a scaffold for cellular support, migration and proliferation, but also acts as the surrounding microenvironment that influences the cellular fate decision by presenting physical and chemical cues as well as binding soluble factors [12]. In vitro, the substrate acts as the primary ECM component, and while a feeder layer of inactivated mouse embryonic fibroblasts (MEF) has been the traditional gold standard, polymeric materials have also been investigated for their ability to support hESC propagation. We have previously reviewed the potential of bio-inspired polymers in determining human stem cell fate, which not only possess the advantage of being a xeno-free culture system but can also be tailored to very specific needs [14].

Early proof of the influence of ECM stiffness on stem cell differentiation was provided by qualitative studies involving mouse mammary epithelial cells that showed increased differentiation when grown on soft gel collagen substrates as opposed to Tissue Culture Plastic [15]. ECM control of stem cell fate by regulating growth factor diffusion has been demonstrated by artificially tethering a growth factor to a substrate, which increased survival of human mesenchymal stem cells (MSCs) [16]. In additional studies, the ECM was also found to be a more potent differentiation cue for MSCs than standard induction cocktails [17]. Tissue-level elasticity has been shown to be able to determine lineage and phenotype commitment in naïve MSCs. Later studies showed that human MSCs could be kept quiescent by growing them on polyacrylamide substrates that mimicked the properties of marrow while preserving their multilineage potential [18]. When NIH/3 T3 cells were cultured on polydimethylsiloxane (PDMS) substrates patterned with varying stiffness, the cells accumulated preferentially on the stiffer regions of the substrates with differential remodelling of ECM on stiff vs. compliant areas, which led to the suggestion that migration, and not proliferation, was responsible [19]. In seminal studies, Engler and colleagues showed that matrices whose elasticities were comparable to brain tissue (“soft matrices”) were neurogenic and stiffer and rigid matrices (with elasticities comparable to muscle and bone tissue, respectively) were respectively myogenic and osteogenic [8]. Substrate compliance was also demonstrated to positively influence survival and functionality of mouse ESC- derived hepatocyte like cells [20].

In this study, we aimed to understand the role of substrate stiffness in two dimensional hESC culture and hoped to devise a PDMS based culture system for directing hESC differentiation. Specifically, we chose to focus on how modulating the mechanical properties of the substrate affected cell density and cell shape, along with maintaining pluripotency and the possibility of lineage specification. hESC (BGO1v) were cultured on commercial Polydimethylsiloxane of varying stiffness both in the presence and absence of basic FGF. Presence of pluripotent cells in culture was determined using Alkaline Phosphatase Assay assay and qPCR was performed at various time points to assess pluripotency and differentiation.

## Results

### Synthesis and Characterization of PDMS Substrates

**Figure 1:**
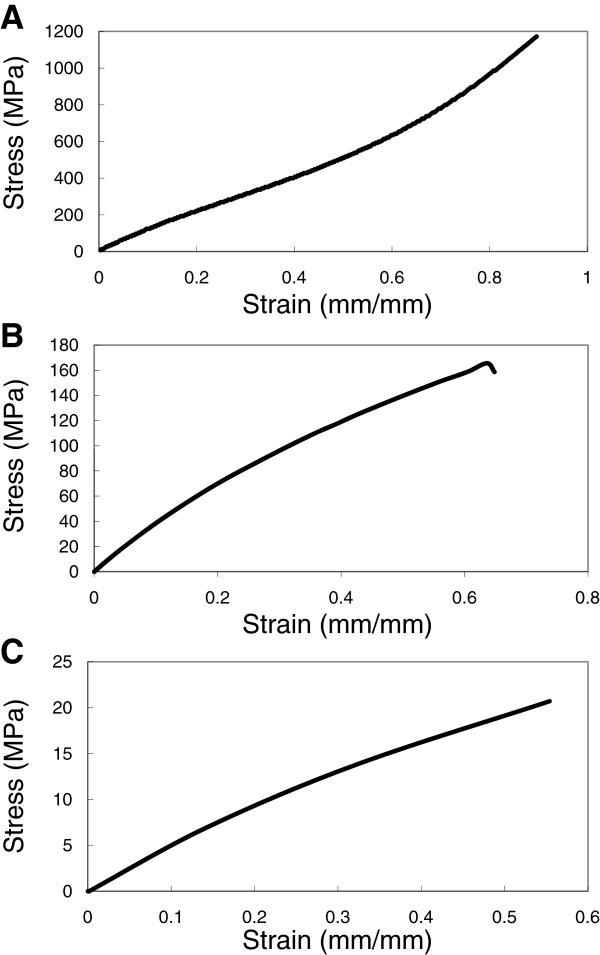
**Testing mechanical properties of the three PDMS formulations used in cell culture studies.** Stress Strain profiles of **A**) 10:1 PDMS; **B**) 20:1 PDMS and **C**) 40:1 PDMS substrates.

**Figure 2:**
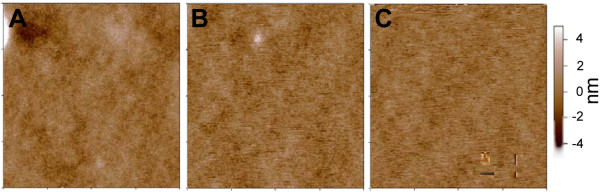
**Surface roughness measured by AFM. Height (Topography) of the three PDMS samples. A**: PDMS 5: 1 RMS roughness: 1.0 nm over 20 micron area **B**: PDMS 10: 1 RMS roughness: 0.8 nm over 20 micron area **C**: PDMS 20: 1 RMS roughness: 0.8 nm over 20 micron area.

Cell culture substrates with varying stiffness were prepared from PDMS by varying the base to cross linking agent ratio from 10:1, 20:1, 40:1. In the following discussion, the various substrates will be referred to by their base: crosslinker ratio e.g. PDMS 10:1. Tangent moduli were calculated from the tensile testing data (Figure 1) and ranged between 0.078 MPa to 1.167 MPa (Table 1). The data for 10:1 PDMS show that the polymer has at least two distinct tangent moduli: lower stiffness was observed at low strain levels than at higher strains. The transition was not observed in 20:1 and 40:1 PDMS, although it is likely that this was because the samples failed before they reached the strain level at which the transition occurs. Surface roughness of the prepared cell culture substrates as determined by tapping mode in AFM was shown to lie between 0.8 nm and 1.0 nm over a 20 μm^2^ area (Figure 2) with no major surface features present. Contact angle measurements (Table 2) indicated that surface hydrophilicity prior to fibronectin treatment decreased with decreasing stiffness of the substrate.

**Table 1:** Tangent moduli of PDMS substrates

**Substrate**	**Tangent Modulus (MPa)**
PDMS 10:1	1.167 ± 0.088MPa
PDMS 20:1	0.397 ± 0.019MPa
PDMS 40:1	0.078 ± 0.008MPa

Moduli calculated from stress- strain curves. Error reported indicate the standard variance from the mean (n = 3).

**Table 2:** Contact angles prior to surface treatment

**Substrate**	**Contact angle (degrees)**
PDMS 5:1	93 ± 1.5
PDMS 10:1	99 ± 1.0
PDMS 20:1	103 ± 0.5

### Cellular attachment, proliferation and morphology

**Figure 3:**
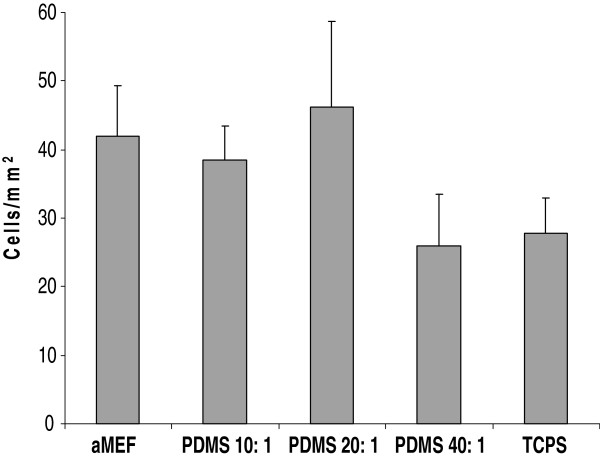
**Cell Density across substrates, independent of cell-cell contact.** Cell density after 12 hours was used as an indicator for degree of cell attachment across various substrates. Error bars represent standard deviation.

**Figure 4:**
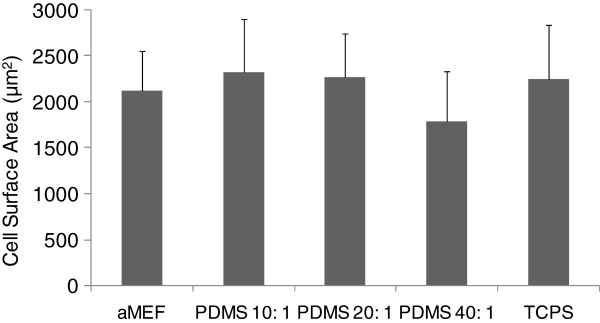
**Cell Morphology across substrates, independent of cell-cell contact.** Cell surface area after 12 hours on various substrates, with 20 cells measured per condition from triplicate samples. Area of cells grown on PDMS 40:1 was significantly lower than that of the other substrates. Error bars represent standard deviation.

Cells grown on PDMS substrates initially proliferated at a rate similar to cells grown on mouse embryonic fibroblasts and fibronectin coated Tissue culture polystyrene (TCPS), which were used as controls. However, after seven days, there were more cells on the various PDMS substrates compared to the TCPS controls, indicating that PDMS could be used as a platform for hESC propagation and differentiation (data not shown). In order to determine that this difference in proliferation was a result of the effect of the substrates on the cells and was not merely a consequence of viable cell attachment, cell densities were measured after 12 hours. The results (Figure 3) indicate that in the early stages, even in the absence of cell- cell contact, cellular attachment was comparable across substrates. Cell shape (also independent of cell-cell contact) and cell surface area was also comparable across substrates at this early time point, with no statistically significant differences with increase in substrate stiffness (Figure 4).

### Self renewal and lineage specification

**Figure 5:**
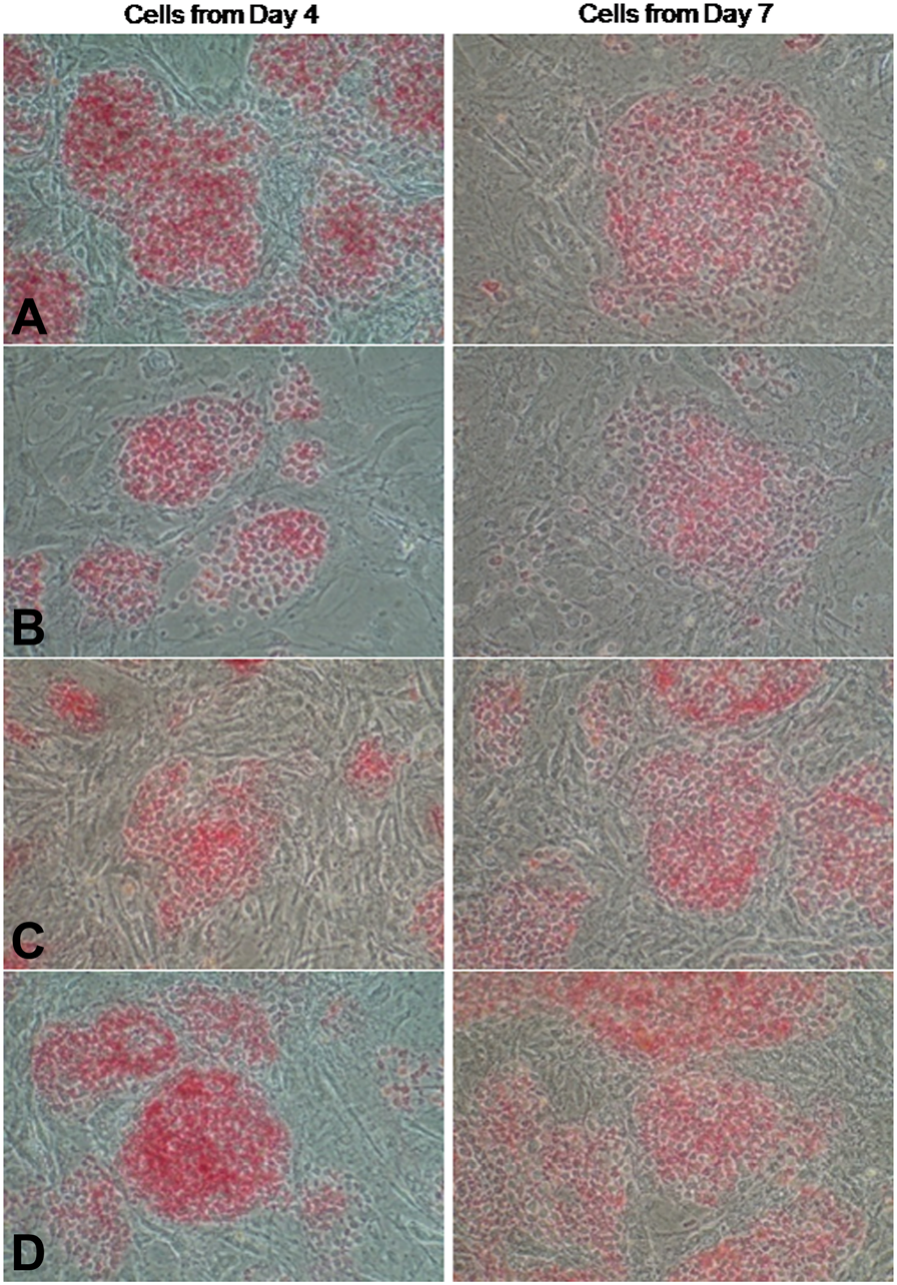
**Alkaline Phosphatase Activity for cells collected from various substrates.** Cells collected from different substrates after 4 and 7 days in culture were re-plated on feeders in the presence of complete hESC medium for four days, following which they were stained for AP activity. **A**: Cells from 10: 1 PDMS, **B**: Cells from 20: 1 PDMS, **C**: Cells from TCPS, **D**: Cells from MEF.

Cells collected from substrates after 4 and 7 days in culture were re-plated onto inactivated MEF layers and cultured in complete hESC medium for 4 days and stained for AP activity. We found that a subpopulation of pluripotent cells remained among the cells cultured on all substrates at Day 4 and at later points (cells collected from substrates on Day 7), while some differentiated cells could be found within most cells stained for AP (Figure 5). These results suggest that these differentiated cells could be early progenitors capable of taking on normal hESC morphology and interacting with cells in a way conducive to proper colony formation.

Gene expression analysis across all substrates at various time points in culture showed that expression of pluripotency markers tended to decrease over time in the absence of basic FGF, as was anticipated. Differential gene expression Expression Index (EI) analysis (Table 3) indicates that across all time points, cells on the various PDMS substrates were more differentiated than those on TCPS. Among the various PDMS substrates, early differentiation appears to follow a stochastic process, with EI values falling sharply between Day 4 and Day 7.

**Table 3:** Expression indices across substrates

**Substrate**	**Day 4**	**Day 7**	**Day 10**
**PDMS 10:1**	23.13 ± 15.82	1.94 ± 0.52	2.02 ± 1.36
**PDMS 20:1**	55.86 ± 9.79	1.98 ± 0.17	0.35 ± 0.08
**PDMS 40:1**	0.36 ± 0.09	2.36 ± 0.41	0.11 ± 0.02
**TCPS**	83.67 ± 38.20	6.44 ± 3.74	7.22 ± 3.69

Expression Index calculated using Equation 1, errors reported are the standard error of the mean (n = 3).

**Figure 6:**
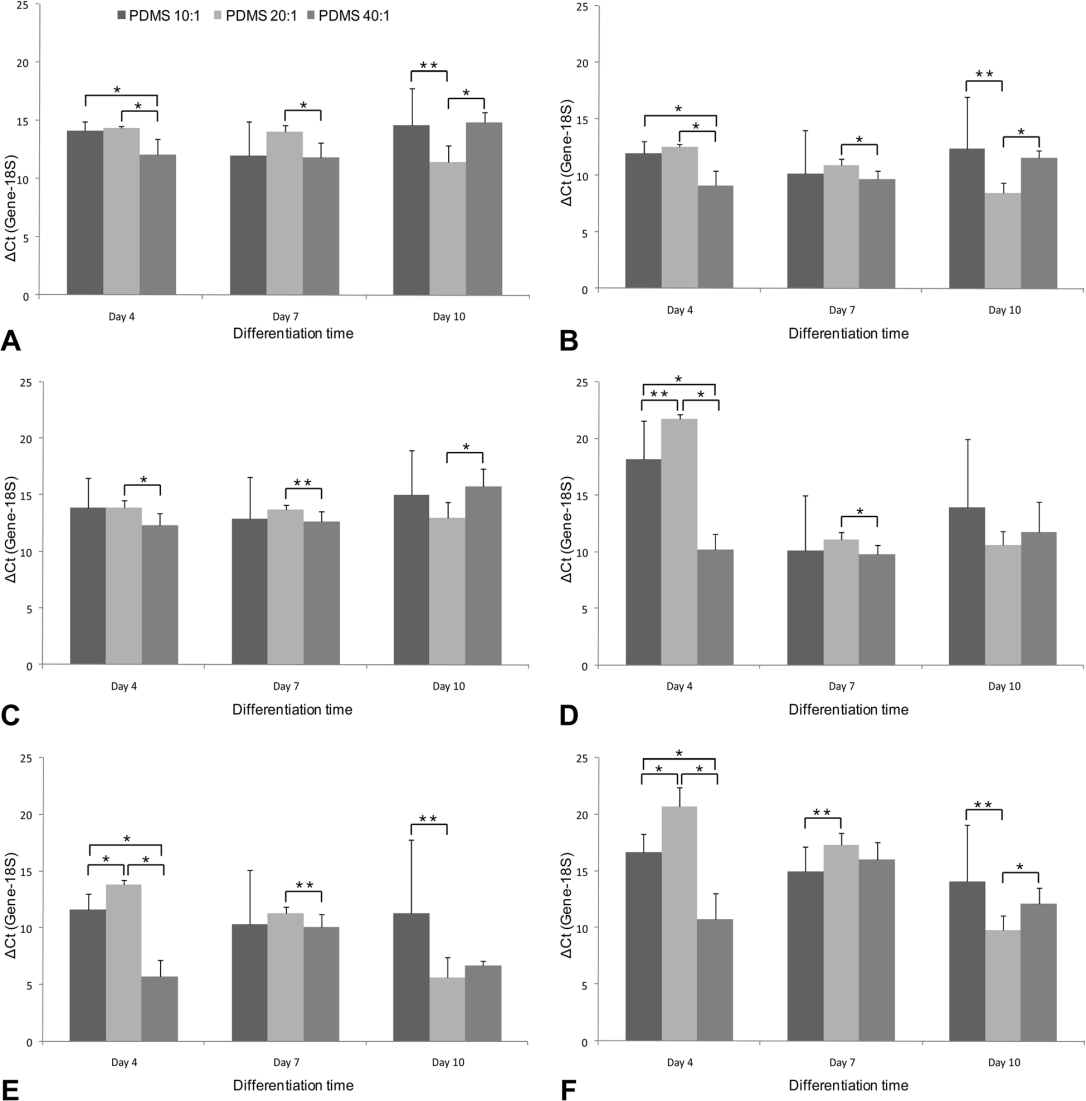
**Comparison of individual gene expression across substrates.** Genes analyzed included **A**: Oct 4 (Pluripotent) **B**: Nanog (Pluripotent) **C**: Sox 2 (Pluripotent) **D**: Neuro **D** (Ectoderm) **E**: IGF2 (Mesoderm) **F**: AFP (Endoderm). (* indicates p < 0.01 and ** indicates p < 0.05).

Across the various PDMS substrates, pluripotency markers decrease over time, with the exception of Nanog in cells cultured on PDMS 20:1(Figure 6A, B, C). Nanog expression peaked at Day 10 on PDMS 20:1 and was significantly higher in cells grown on this substrate than the other two at this time point. Expression of the three representative differentiation markers tested (NeuroD, IGF2, AFP)increased on almost all PDMS substrates from Day 4 to Day 7, with change in NeuroD expression being the most, followed by that of AFP. Few exceptions included IGF2 expression on PDMS 40:1 and AFP expression on PDMS 40:1. On all PDMS substrates, NeuroD expression peaks at Day 7 but by Day 10 falls to slightly lower levels, while still remaining higher than Day 4 (Figure 6D). NeuroD expression on the softest substrate was significantly higher than the other two at this early time point. IGF2 and AFP expression show a consistent increase with time, but were the highest on PDMS 20:1 (Figure 6E, F).

## Discussion

We have shown here that substrate stiffness affects cellular spreading, proliferation and gene expression of hESCs. PDMS was chosen as the substrate because it is easy to handle, inexpensive, does not swell in contact with water and can be micropatterned using techniques such as soft lithography. Surface treatments with UV radiation or ethanol, such as those used here; also do not affect material properties [21]. Other properties of PDMS, such as thermal stability, transparency and chemical inertness, make it particularly useful in bioengineering, in spite of potential batch to batch variabilities [22], [23]. We were able to fabricate substrates with stiffness varying from 0.078 to 1.167 MPa, a range similar to that reported by others [24,25]. We generated even stiffer substrates by using a base: crosslinker ratio of 5:1 (PDMS 5:1) and performed initial characterization studies but decided to focus on softer substrates for cell culture and gene expression analysis. However, even within the given range of stiffness tested, substrate mediated biological effects were observed. Although proliferation increased on substrates, cell spreading did not increase with increased stiffness. These results are in direct contrast with previously reported studies conducted with terminally differentiated cells [26,27]. Our results tend to indicate that hESCs might react differently to substrate stiffness when compared with terminally differentiated cells like fibroblasts or endothelial cells [26,27]. These differences in cellular behaviour merits further studies in determining how cells in their undifferentiated state respond to substrate stimuli. Cells on stiffer substrates tend to exert larger traction forces [28] and it may be hypothesised that substrates affect cell development by affecting cellular migration and movement via the dynamics and size of adhesion sites. Substrate compliance are a major factor in cell culture studies and the probable molecular responses to these substrates have been discussed in detail in literature [28,29].

Our data indicate that at early time points, stiffness mediated differentiation follows a rather stochastic process, but certain trends begin to appear with longer duration in culture (Table 3). By Day 10, the mesodermal marker IGF2 was found to be the most highly expressed gene, with its expression being the highest on PDMS 20:1, one of the stiffer substrates. The dynamics of differentiation across different substrates reveals interesting trends. Specifically, the PDMS 20:1 demonstrates stronger dynamics in AFP and IGF2 expression (Figure 6E, F), while other conditions do not show such an obvious trend in differentiation. One possible hypothesis for the mechanism of this increased AFP and IGF2 expressions correlated with stiffness is that stiffer substrates provide an environment that more closely mimics those experienced by migrating mesodermal and endodermal cells in the early embryo. Our data also indicates that there is greater increase in IGF2 (mesoderm) expression (Figure 6E), when compared with AFP (endoderm) expression (Figure 6F), indicating a greater propensity for increased mesodermal differentiation on PDMS 20:1 substrate. At this juncture, it is also interesting to note that MSCs - which originate from the mesoderm - respond highly to substrate stiffness based cues for lineage specification*.* Recent studies involving hESCs cultured in three dimensional polymeric substrates with a broad range of elasticities also indicated that as stiffness increased, mesodermal differentiation was favoured over endodermal lineages [30].

While the molecular mechanisms linking substrate stiffness and hESC differentiation still remain to be explored, we can speculate this to be either a direct effect of mechanical properties of the substrate on cellular differentiation events, or an indirect effect related to the changes in cell spreading and migration. Thus the stiffness mediated increase in cell attachment might be mimicking the environment of migrating mesoderm cells, thereby supporting the growth and differentiation of more adhesive cells. We also need to take into account that altering substrate rigidity also affects the overall chemical composition of the substrate which could affect differentiation. However, in previous studies on polyacrylamide gels of different formulations but similar stiffness, cell morphology remained similar [26].

One caveat to keep in mind is that not all mechanosensitive cell types respond similarly to changes in substrate stiffness [29]. Multiple tissues may have similar elasticities, and cell types respond differently to mechanical signals, in a manner somewhat similar to what they experience in their native tissue [27].

## Conclusions

In this study, we have demonstrated that fibronectin-coated PDMS substrates are capable of supporting hESC attachment and proliferation. We have shown here that substrate stiffness affects proliferation, while cell spreading and cell attachment remains comparable across the range of stiffness tested. With increased time in culture, differentiation increased and gene expression associated with mesodermal differentiation was upregulated as stiffness increased from soft to stiff, suggesting that the substrate is an important variable that needs to be carefully considered in developing protocols for stem cell propagation and differentiation. Future studies will focus on whether such a system can be used to bring about terminal differentiation of hESCs towards defined cell types of the mesodermal lineage.

## Materials and methods

### ESC culture

A variant hESC line (BG01v) was cultured on MEF feeders that have been inactivated with mitomycin-C. Cells were cultured in hESC medium, which consisted of Dulbecco's modified Eagle's medium (DMEM)/F12 medium (Gibco, Cat. No. 11320–033) supplemented with 20% knockout serum replacement (KSR; Gibco, Cat No. 10828–028), 1 mM L-glutamine (Gibco, Cat. No. 25030–081), 0.1 mM minimal essential medium nonessential amino acids (Gibco, Cat. No. 11140–050), 50 U/ml penicillin and 50 μg/ml streptomycin (Gibco, Cat. No. 15070–063), 4 ng/ml basic fibroblast growth factor (Sigma, Cat. No. F-0291), and 0.1mM β-mercaptoethanol (Sigma, Cat. No. M7522). Cells were passaged every 3–5 days by treatment for 2–3 minutes with 1 mg/ml collagenase type IV (Gibco, Cat. No. 17104–019) in ES cell medium, followed by a 40 second treatment with 0.05% trypsin. Cells were plated at 50,000 cells/35 mm plate and grown at 37°C and 5% CO_2_.

### Substrate synthesis and characterization

The PDMS substrate was prepared from the commercially available Sylgard 184 silicone elastomer kit (Dow Corning, Midland, MI) by mixing the base and the curing agent in varying ratios. Specifically, PDMS with base: crosslinker w/w ratio 10:1, 20:1, and 40:1 were prepared. The pre-polymer mixtures were mixed thoroughly for at least 5 minutes, degassed, and poured into 35 mm polystyrene tissue culture Petri dishes. PDMS was then cured for at least 60 hours at 22-33°C. Samples were stored at room temperature in a vacuum desiccator.

Tensile testing was done to characterize the bulk mechanical properties of the substrate. Specifically, 1 mm dog-bone shaped strips were subjected to a tensile load at a strain rate of 10 mm/min and the test was conducted to failure. The elastic modulus was determined manually by calculating the slope of the stress strain curve within linear limits.

Topographical and phase images were taken on an MFP-3D AFM (Asylum Research, Santa Barbara, CA). Images were obtained in non-contact (AC/tapping) mode and height, amplitude and phase images were taken using a silicon cantilever (AC-240 TS, Olympus Instruments) at a scan speed 1 Hz at 512 pixels/line. The scan size was 20 μm × 20 μm.

Surface wetting properties of the various substrates were evaluated by measuring the static water contact angles via the sessile drop method using a Ramé-Hart Goniometer/ Tensiometer (Model 500) equipped with a special optical system and a CCD camera and the image was analyzed using DROPImage Advanced for contact angle determination.

### Cell culture on PDMS substrates

Before conducting cell culture experiments, PDMS substrates were sterilized by treating them with ethanol under ultraviolet light for 1 hour, followed by a second round of UV exposure for another 30 minutes. To promote cell attachment to the various substrates studied, plates coated with various formulations of PDMS and the polystyrene plates were treated with 2.5 mL of 10 μg/mL of human plasma fibronectin (Chemicon, Cat. No. FC010) overnight at 37°C. Substrates were then washed twice with PBS and cells were seeded at 50,000 cells per 35 mm plate. To promote differentiation, cells were grown in differentiation medium (hESC medium without bFGF). Cells grown on inactivated MEF feeders in hESC medium were used as controls. Medium was changed on the second day and on every following day. On the 4^th^, 7^th^, and 10^th^ day cells were collected from the plates by treating them with collagenase and trypsin as described above. Cells that remained attached following enzymatic passaging were collected using a rubber cell scraper. Cell counts were performed using a hemocytometer. The collected cells were snap frozen in liquid nitrogen and stored at -80°C for subsequent gene expression analysis. All experiments were performed with three replicates per condition.

### Alkaline phosphatase activity

Some of the collected cells were also seeded onto plates with inactivated MEF feeders and propagated in hESC medium for 4 days after which an Alkaline Phosphatase Substrate Kit (Vector Labs, Cat. No. VC-SK-5100-KI01) was used to assay for alkaline phosphatase (AP) activity.

### Cell surface area calculations

To study how substrates affect morphology independent of cell-cell contact, cells were plated at a density of 10,000 cells per 35 mm plate and grown in hESC growth medium. Cells seeded at the same density on acellularized MEF layers were used as controls. After 12 hours, images of cells from different experimental conditions were captured using a Nikon Eclipse TS100 inverted microscope and a Nikon Coolpix 5000 digital camera. Area was calculated using ImageJ software by manually outlining the cell perimeter with each area measurement performed twice. Cell density was calculated by manually counting the number of cells in the field of vision and extrapolating to the cell density in each of the 35 mm dishes. Each condition was performed with three replicate plates, and images of multiple cells were captured from each plate. All cells that were in contact with other cells were excluded from the analysis.

### Gene expression analysis

For gene expression analysis, samples were prepared by isolating total RNA using TRIZOL (Invitrogen, Cat. No. 10296–010, Carlsbad, CA) according to manufacturer's instructions. Briefly, cell pellets were treated with TRIZOL and chloroform, RNA from the aqueous phase was precipitated in isopropyl alcohol, washed with 75% ethanol, and dissolved in water. RNA was quantified using a UV–vis Spectrophotometer (Biomate 3, Thermo Scientific, Waltham, MA). cDNA was reverse-transcribed from 1 μg of total RNA according to manufacturer's protocols using the High Capacity cDNA Reverse Transcription Kit (Applied Biosystems, Cat. No. 4368814 Foster City, CA). The reactions were incubated for 10 minutes at 25°C and for 120 minutes at 37°C.

Expression of pluripotent and differentiation genes was analyzed using quantitative real time RT-PCR using Taqman primers (Applied Biosystems, Foster City, CA); performed in an ABI HT7900 system (Applied Biosystems, Foster City, CA) and the data were acquired using sequence detection system software (SDS v2.2.1, Applied Biosystems, Foster City, CA). The original replicates (n = 3) for each condition were tested in duplicate, and all failed reactions (termed “undetermined” by the software) were excluded from the analysis. ΔCt values were obtained by normalizing the Ct values against the endogenous 18S ribosomal RNA. Data analysis for differential expression between the different samples was conducted in triplicate and Student’s t-test was conducted to ascertain the significance of differential expression.

Expression Index was used to detect the relative differentiation state of cells and was based on average Ct values from triplicate measurements and used the mathematical model described in Noaksson et al. [31] wherein EI is given by the equation

$$\mathrm{EI}=K_{\mathrm{RS}}\frac{\sqrt[{m}]{{\left(1+E_{\mathrm{gene}1m}\right)}^{\mathrm{Ctgene}1m}.{\left(1,+,E_{\mathrm{gene}2m}\right)}^{\mathrm{Ctgene}2m}\ldots {\left(1+E_{\mathrm{genem}}\right)}^{\mathrm{Ctgenem}}}}{\sqrt[{n}]{{\left(1+E_{\mathrm{gene}1n}\right)}^{\mathrm{Ctgene}1n}.{\left(1,+,E_{\mathrm{gene}2n}\right)}^{\mathrm{Ctgene}2n}\ldots {\left(1+E_{\mathrm{genen}}\right)}^{\mathrm{Ctgenen}}}}$$

where E is the PCR efficiency, Ct is the threshold cycle, m and n are the number of genes that are upregulated and downregulated, respectively, upon hESC differentiation. K_RS_ is the relative sensitivity constant (accounts for the differences in fragment lengths of templates) was not determined since it does not affect the relative comparison of samples.

## Competing interests

The authors declare that they have no competing interests. There is no conflict of interest in the reporting of this data by any author.

## Acknowledgements

We would gratefully like to thank Dr. Jennifer Wayne for granting access to the mechanical testing systems in her laboratory and for help with analyzing mechanical testing data. Funding for this work was provided in part by grant EEC-0234104 from the NSF/NIH Bioinformatics and Bioengineering Summer Institute Program and NSF-CAREER 074556 (RRR). Additional funding was provided by the VCU Honors Summer Undergraduate Research Program (NE).

## Authors’ contributions

NE fabricated the substrates and performed cell culture and characterization experiments. RR performed cell culture experiments, gene expression analysis and drafted the manuscript. VY performed the AFM and contact angle measurements. RRR is the principal investigator and was responsible for all elements of this research. All authors have read and approved the final manuscript. No writing assistance was used in the production of this manuscript.
